# Ultraviolet-visible light spectral transmittance of rabbit corneas after riboflavin/ultraviolet-A (365 nm) corneal collagen cross-linking

**Published:** 2013-10-04

**Authors:** Ho Sik Hwang, Man Soo Kim

**Affiliations:** Department of Ophthalmology & Visual Science, College of Medicine, Catholic University of Korea

## Abstract

**Purpose:**

To determine the effect of riboflavin/ultraviolet-A (365 nm) corneal collagen cross-linking on the transmission of the ultraviolet-visible (UV-VIS) light spectrum through the cornea.

**Methods:**

Twelve New Zealand white male rabbits were used in this research. Cross-linking was performed unilaterally on the right eyes of the animals while only the epithelium was removed on the left eyes as the control. Seven weeks after cross-linking, the animals were euthanized, and the enucleated eyes were processed for transmission spectroscopy. To confirm that the cross-linking procedures was done successfully on the right corneas, the tensile force-extension relationship was measured using six corneas from three of the rabbits after the transmission spectrum was determined.

**Results:**

Seven weeks after cross-linking, ten of the 12 rabbits had clear corneas in the cross-linked and control eyes. The two rabbits with neovascularization and granular opacities in the right corneas were not included in subsequent measurements. In the cross-linked corneas, transmittance was 87.57% at 650 nm, and decreased continuously as the wavelength shortened. From 315 nm, the transmittance rapidly decreased and was 35.52% at 300 nm. In the control corneas, transmittance was 95.95% at 650 nm and decreased continuously as the wavelength shortened. Below 315 nm, the transmittance rapidly decreased, to 40.29% at 300 nm. The transmittance of the cross-linking corneas was 10%–20% lower than that of the control corneas. The difference was 8.38% at 650 nm and increased as the wavelength shortened, reaching a maximum of 20.59% at 320 nm, and decreased rapidly to 4.77% at 300 nm. The tensile force-extension relationship showed that a greater force was necessary to extend the cross-linking corneas over 500 µm than that of the control corneas.

**Conclusions:**

The transmittance of the cross-linked corneas was 10%–20% lower than that of the control corneas. The difference increased as the wavelength decrease, reaching a maximum at 320 nm and then decreasing rapidly. Ultraviolet collagen cross-linking exhibited a protective effect against ultraviolet penetration.

## Introduction

Riboflavin (vitamin B_2_)/ultraviolet-A (UVA; 365 nm) corneal collagen cross-linking is a treatment option for keratoconus [1-8] or cornea ectasia [7]. The following describes the procedure. Corneal epithelia are removed, and riboflavin penetrates the corneal stroma. The cornea is then irradiated with UVA 365 nm, which produces reactive oxygen species (ROS), such as singlet oxygen and superoxide anion radicals, which induce the covalent bonds between the collagen proteins [4]. This collagen cross-linking leads to biomechanical stabilization of the cornea [9]. Subsequently, the stiffness of the cornea increases, and the progression of keratoconus or cornea ectasia is thus hampered or stopped.

The corneal stroma is unique in that it possesses a homogeneous distribution of small diameter (25 to 30 nm) fibrils that are regularly packed within lamellae. It was hypothesized, based on the physical properties of light, that this lattice-like structure produces minimal light scattering and, therefore, transparency to the visible spectrum [10-12]. However, regarding the ultraviolet spectrum (UVA: 400 to 315 nm, UVB: 315 to 280 nm, UVC: 280 to 100 nm), corneal transmittance decreases below 400 nm, and below 300 nm is nearly zero [13,14]. The cornea plays a key role in absorbing UV light and in protecting the inner eye such as the crystalline lens and retina against oxidative injury.

We hypothesized that if collagen cross-linking changed the inner structure of the corneal stroma, including the collagen fibrils and their arrangement, then ultraviolet-visible (UV-VIS) light transmittance through the cornea could be altered. Ghanem et al. reported that after cross-linking for treating pseudophakic bullous keratopathy, the corneal thickness decreased, and the cornea became clear, although this condition was temporary [15]. In contrast, Cejka et al. reported that throughout the measurable UV-VIS spectral range, a UVB-irradiated cornea absorbs more light than a normal cornea [16].

If UV light transmittance increases after cross-linking, the incidence of cataract or retinal complications (e.g., age-related macular degeneration) increases. Moreover, since most patients undergoing cross-linking are young, the long-term effects on the inner eye are more profound. If VIS light transmittance decreases significantly, the corrected visual acuity is reduced. Therefore, the change in the UV-VIS light transmission spectrum after cross-linking is an important issue. However, to our knowledge, no reports regarding this important factor have been published.

In this study, we performed in vivo cross-linking on corneas in rabbits. After 7 weeks, we determined the effect of riboflavin (vitamin B_2_)/UVA 365 nm corneal collagen cross-linking used to slow the progression of keratoconus or cornea ectasia on the transmission of the UV-VIS light spectrum through the cornea.

## Methods

### Animals

This study was approved by the Animal Experimentation Committee of the Catholic University of Korea. Animal experiments were conducted in compliance with the Statement of the Association for Research in Vision and Ophthalmology on the Use of Animals in Ophthalmic and Vision Research. Twelve New Zealand white male rabbits (numbered #1 to 12) weighing 2.5–3.0 kg were used in this research. Cross-linking was performed unilaterally on the right eyes of the animals while only the epithelium was removed on the left eyes, as the control. Seven weeks after cross-linking, the animals were euthanized, and the enucleated eyes were processed for transmission spectroscopy.

### Cross-linking procedure

The animals were anesthetized with a mixture of 7.5 mg/kg tiletamine hydrochloride, 7.5 mg/kg zolazepam hydrochloride (Zoletil; VIRBAC Laboratories, Carros, France) and 5 mg/kg xylazine hydrochloride (Rompun; Bayer HealthCare, Leverkusen, Germany) before treatment. Cross-linking was performed unilaterally on the right eyes of the animals, whereas only the epithelium was removed on the left as the control. The protocol used was similar to the technique reported by Wollensak et al. [17]. Briefly, 20% alcohol was applied to the cornea with an 8.5-mm well (the same procedure as for laser-assisted subepithelial keratectomy) to disturb the tight epithelial junctions. The epithelium was carefully removed with a hockey knife. After epithelial abrasion, the cornea was moistened for 10 min with 0.1% riboflavin solution (10.95 mg riboflavin-5-phosphate in 10 ml of 20% dextran T-500 solution [Peschke GmbH, Nurnberg, Germany]). During the next 30 min, UVA light at 365 nm with 3 mW/cm^2^ surface irradiance was projected onto the central cornea using a UV-X irradiation system (Peschke). Riboflavin drops were reapplied topically to the corneal surface every 5 min during UVA irradiation. Levofloxacin (Cravit; Samil, Seoul, Korea) and fluorometholone (Ocumetholone; Samil) drops were applied five times daily for 7 days after cross-linking.

### Transmission spectroscopy

Seven weeks after cross-linking, the animals were euthanized with an intravenous overdose of sodium pentobarbital (>100 mg/kg, noninhaled agent, ear vein), and the enucleated eyes were processed for transmission spectroscopy. Initially, with the naked eye and the globe in situ, the presence of any abnormalities, such as ulcers or corneal opacity, was determined, and then photographs were taken with a digital camera. The central cornea thickness was measured with an ultrasonic pachymeter (SP-3000, TOMEY, Phoenix, AZ) three times with the values averaged. The eyeball was enucleated, and the corneoscleral button was trephined. The corneoscleral button was placed, endothelial side up, on a cutting block, and then punched from the endothelial side, using an 8.0-mm Hessburg-Barron punch (Katena, Danville, CA).

**Figure 1:**
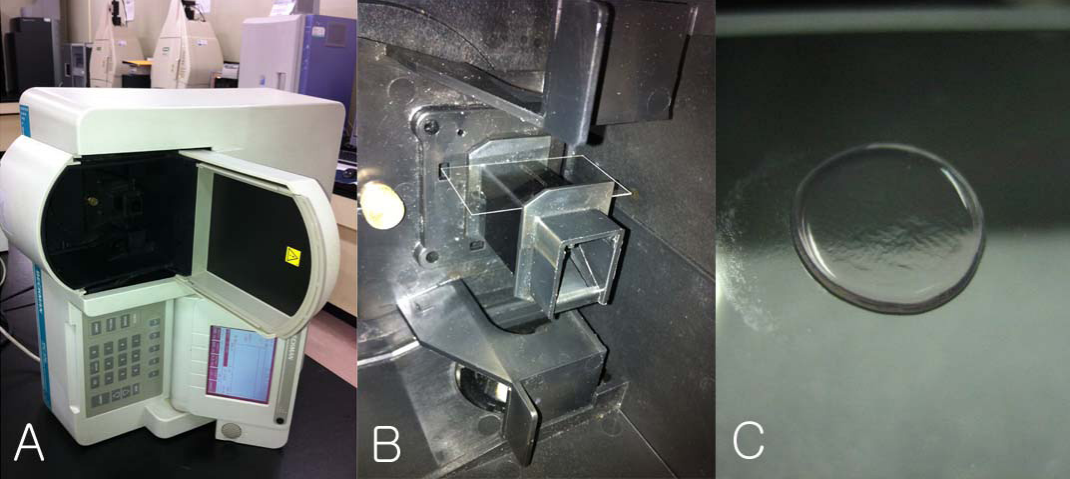
A spectrophotometer used for measuring the ultraviolet-visible light transmittance spectrum. **A**: The spectrophotometer was set up so that the cornea and microscope cover glass could be placed perpendicularly to the measuring beam. **B**: The cover glass was placed on the cuvette holder. **C**: The corneal sample was placed on the cover glass with the epithelium directed downward.

To measure the UV-VIS transmittance spectrum, a spectrophotometer (DU-530 Life Science UV/Vis Spectrophotometer, Beckman Coulter, Indianapolis, IN) was used. The spectrophotometer was set up so the cornea and microscope cover glass (Deckglaser, Lasec, South Africa) could be placed perpendicular to the measuring beam (Figure 1A). The cover glass was placed on the cuvette holder (Figure 1B). The scan range was set at 300–650 nm (5 nm intervals), which included ultraviolet and visible light. The “transmission percentage” indicator was chosen. First, the base spectrum of the microscope cover glass was measured. Second, the corneal sample was placed on the cover glass with the epithelium directed downward. By placing the samples in this orientation, the measuring light beam of the spectrophotometer entered the cornea from the epithelial side, that is, from the same direction as the cornea in situ. The cover glass and the cornea were replaced. Their position was adjusted so that the rectangular measuring beam was located in the center of the cornea. After adjustments were made, the cornea samples were scanned.

### Measurement of corneal stiffness

**Figure 2:**
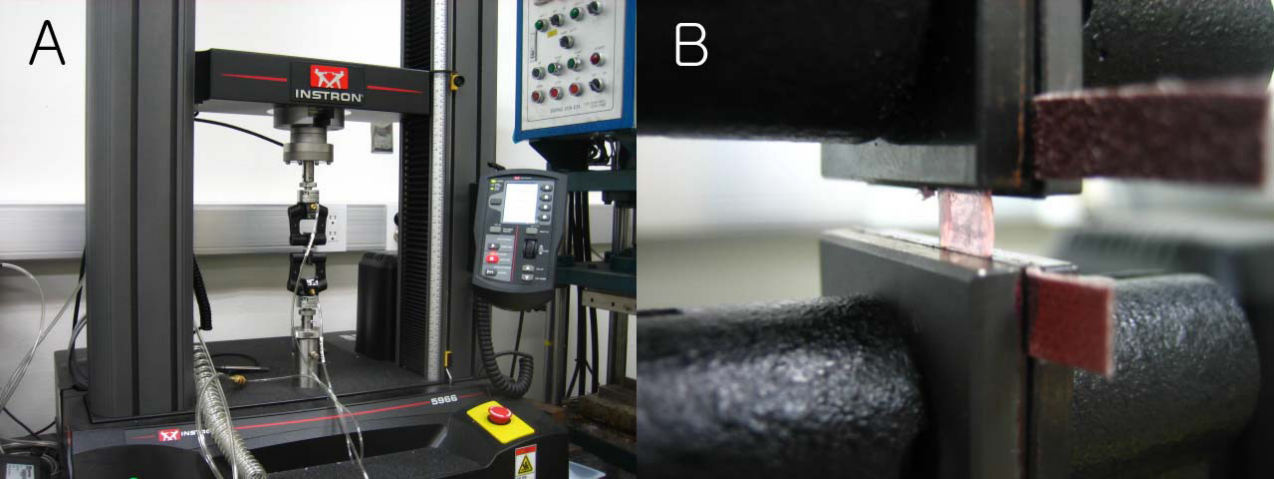
Measurement of corneal stiffness. **A**: A stress-strain curve was obtained using the Biomaterial Universal testing machine. **B**: The 8.0-mm diameter corneas were clamped 2.0 mm on each side.

To confirm that the cross-linking procedures were performed successfully on the right corneas, the tensile force-extension relationship was measured using six corneas from three rabbits after the transmission spectrum was determined. We compared the three right and left corneas. The tensile force-extension relationship was measured using the Biomaterial Universal testing machine (Instron Model 5966, Illinois Tool Works, Grove City, PA; Figure 2A) at room temperature. Ultrasound moisture was used to keep the specimens moist. The 8.0-mm diameter corneas were clamped 2.0 mm on each side (Figure 2B). The distance between the clamps was 4 mm. Force and length data were collected automatically by the computer. The specimens were loaded under a constant velocity of loading (10 mm/min) for one cycle.

### Histological evaluation

To confirm that cross-linking procedures were performed correctly on the right corneas, six eyes from three rabbits were processed for histologic evaluation after transmission spectroscopy. They were fixed with 4% buffered paraformaldehyde and then stained with hematoxylin and eosin (H&E).

### Terminal deoxynucleotidyl transferase-mediated dUTP nick end labeling assay

We assessed keratocyte apoptosis after cross-linking using terminal deoxynucleotidyl transferase-mediated dUTP nick end labeling (TUNEL) assay*.* Tissue sections were removed from paraffin blocks of corneas. A fluorescence-based TUNEL assay was used according to the manufacturer’s instructions (In Situ Cell Death Detection Kit, Cat No: 11 684 809 910; Roche Applied Science, Manheim, Germany). Photographs were obtained with a Zeiss LSM (Jena, Germany) 510 Meta confocal microscope.

## Results

**Figure 3:**
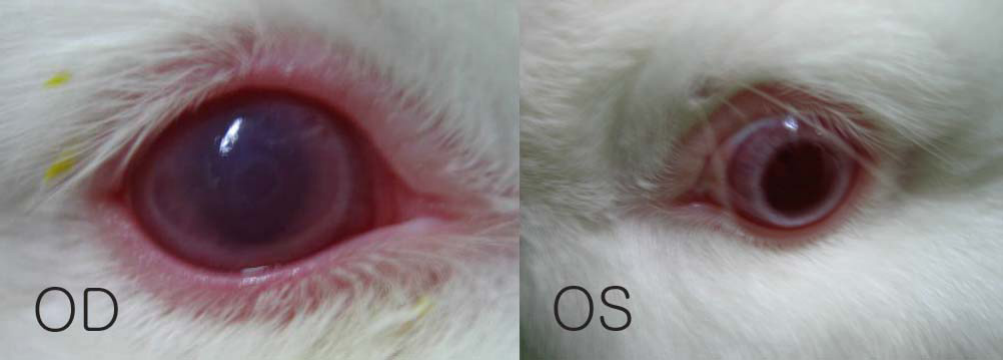
Corneal edema after cross-linking. A right cornea (OD) 2 days after cross-linking showing severe edema and opacity compared to the control cornea (OS). At approximately 1 week after cross-linking, corneal edema was not observed with the naked eye.

Figure 3 shows a right cornea with severe edema and opacity 2 days after cross-linking. Subsequently, however, the corneal edema decreased. At approximately 1 week after cross-linking, corneal edema was not observed with the naked eye.

**Figure 4:**
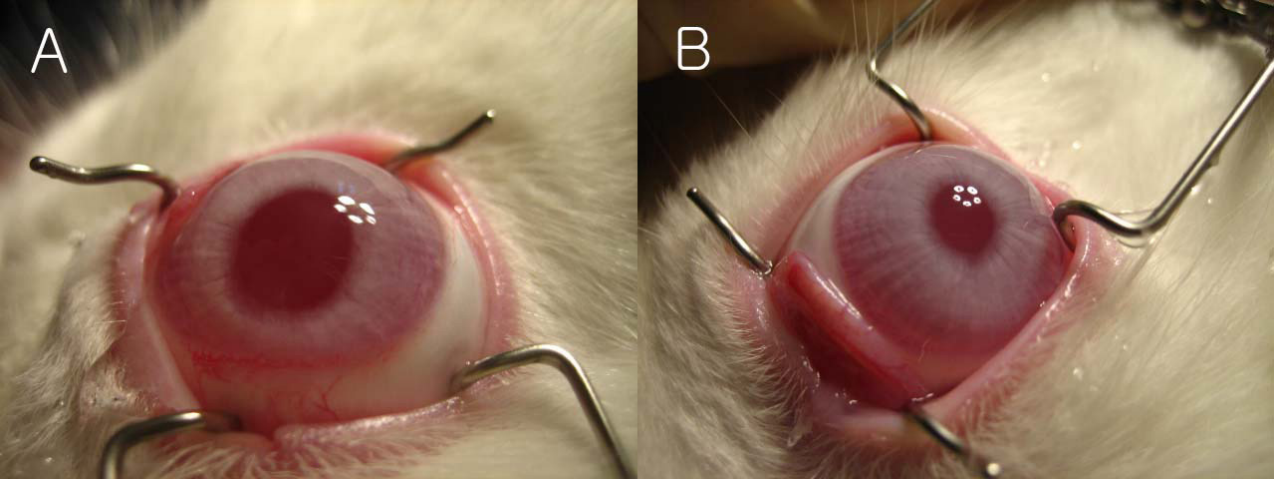
Photographs of corneas before enucleation, 7 weeks after cross-linking. Ten of 12 rabbits exhibited clear corneas in the cross-linked (**A**) and control eyes (**B**).

**Figure 5:**
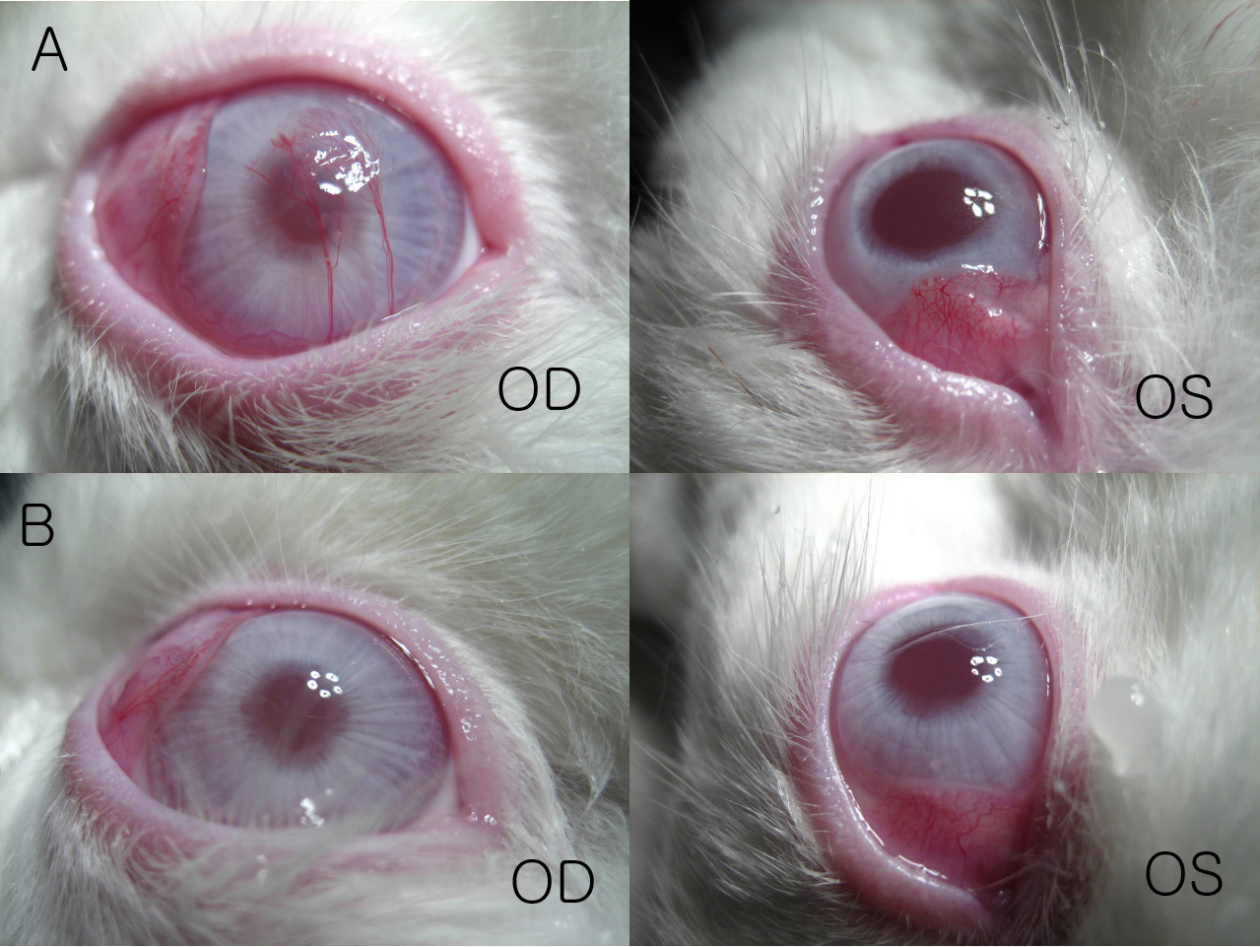
Complications after cross-linking. **A**: In one rabbit (#3), corneal neovascularization was found at the center of the right cornea. **B**: In another rabbit (#5), granular-type opacities were observed at the center of the right cornea. The left corneas of both rabbits were clear.

Figure 4 shows photographs of the corneas before enucleation, 7 weeks after cross-linking. Ten of the 12 rabbits exhibited clear corneas in the cross-linked eyes (Figure 4A) and the control eyes (Figure 4B). In one rabbit (#3), corneal neovascularization was found at the center of the right cornea (Figure 5A). In another rabbit (#5), granular-type opacities were observed with the naked eye at the center of the right cornea (Figure 5B). The left corneas of both rabbits were clear. The two rabbits with neovascularization and granular opacities were not included in the subsequent measurements.

Table 1 shows the central corneal thickness measured before enucleation, 7 weeks after cross-linking. The average central cornea thickness of the cross-linked cornea was 367 µm and 394 µm for the control cornea. The cross-linked cornea was 27 µm thinner than that of the control cornea. However, in three of ten rabbits (#6, #9, and #11), the difference in thickness between the right and left corneas was less than 7 µm.

**Table 1:** Central corneal thickness of cross linking corneas and control corneas

Rabbit		#1	#2	#4	#6	#7	#8	#9	#10	#11	#12	Average
Central corneal thickness (µm)	Cross-linking cornea	328	357	396	365	336	368	382	386	400	330	367
	Control cornea	361	409	415	363	369	414	384	408	407	375	394
	Difference	33	52	19	−2	33	46	2	22	7	45	27

**Figure 6:**
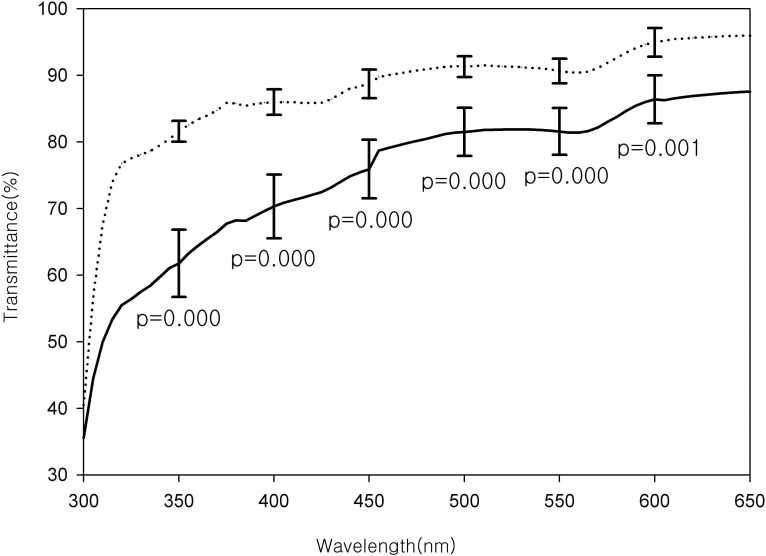
The average transmission spectra of ten right (cross-linking, solid line) and left (control, dashed line) eyes from ten rabbits. The transmittance of the cross-linked corneas was 4.77%–21.28% lower than that of the control corneas. The error bars mean the standard deviation of the transmission percentage between animals in each group. In general, the individual variation among the cross-linked corneas was larger than that among the control corneas.

Figure 6 shows the average transmission spectra of ten right and left eyes from ten rabbits. In the cross-linked corneas, transmittance was 87.57% at 650 nm, and decreased continuously as the wavelength shortened. From 315 nm, the transmittance rapidly decreased and was 35.52% at 300 nm. In the control corneas, transmittance was 95.95% at 650 nm, and decreased continuously as the wavelength decreased. Below 315 nm, the transmittance rapidly decreased, to 40.29% at 300 nm. The transmittance of the cross-linked corneas was 10%–20% lower than that of the control corneas. In general, the individual variation among the cross-linked corneas was larger than that among the control corneas (Table 2).

**Table 2:** Average and standard deviation of transmittance spectrum of cross linked cornea and control cornea

Wavelength		300 nm	350 nm	400 nm	450 nm	500 nm	550 nm	600 nm	650 nm
Cross linked cornea	Average (%)	35.515	61.776	70.304	75.919	81.518	81.574	86.404	87.569
	Standard deviation (%)	3.707	5.036	4.784	4.405	3.619	3.527	3.598	3.688
Control cornea	Average (%)	40.285	81.598	85.973	88.71	91.297	90.667	94.942	95.952
	Standard deviation (%)	3.953	1.573	1.93	2.139	1.572	1.851	2.151	2.487

**Figure 7:**
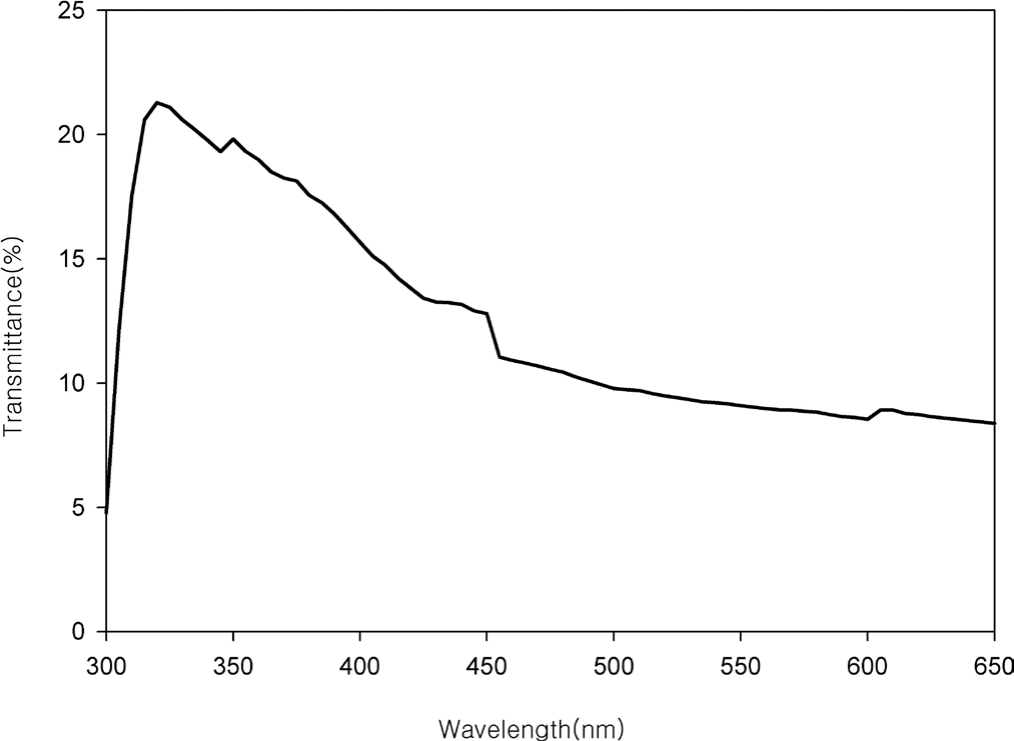
The difference between the transmittance of the control corneas and that of the cross-linked corneas from ten rabbits. The difference was 8.383% at 650 nm and increased as the wavelength decreased, reaching a maximum of 20.591% at 320 nm, and decreasing rapidly to 4.770% at 300 nm. Additionally, a local hump was found between 425 and 455 nm, and a focal maximum at 350 nm.

Figure 7 shows the difference between the transmittance of the control corneas and that of the cross-linked eyes from ten rabbits. The difference was 8.38% at 650 nm and increased as the wavelength decreased, reaching a maximum of 20.59% at 320 nm and decreasing rapidly to 4.77% at 300 nm. Additionally, a local hump was found between 425 and 455 nm and a focal maximum at 350 nm.

**Figure 8:**
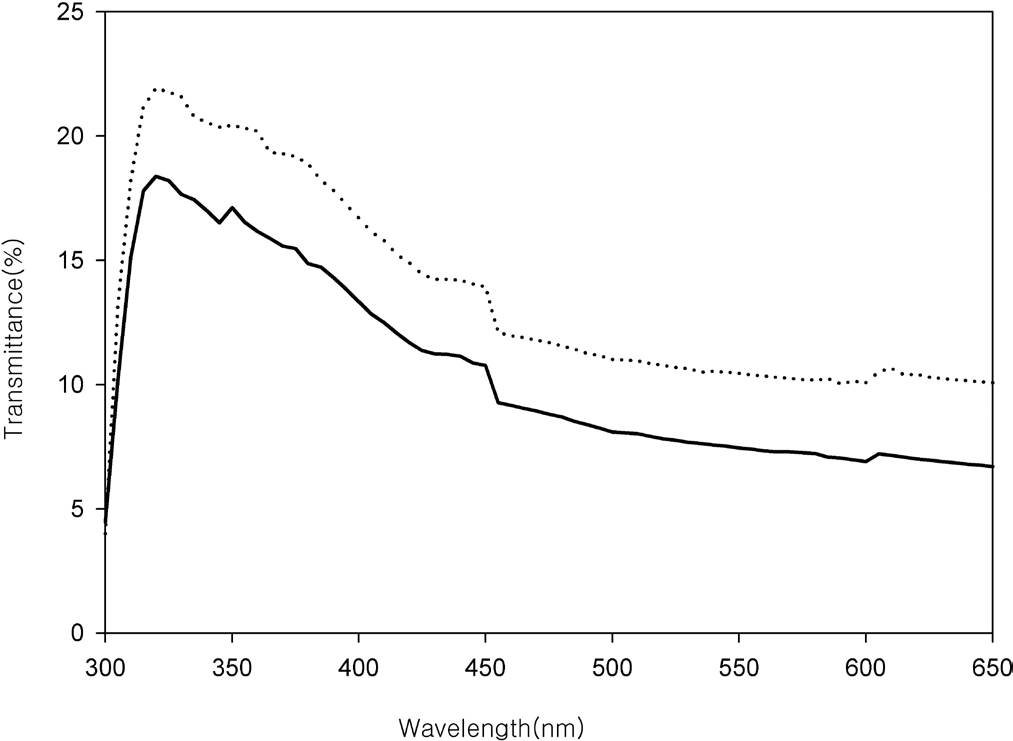
The difference between the transmittance of the control corneas and that of the cross-linked corneas of three rabbits (#6, #9, and #11) and the other seven rabbits. The solid line represents those whose corneal thickness differences were less than 7 µm (#6, #9, and #11), and the dashed line represents the remaining rabbits. The transmittance difference of the three rabbits was 3%–4% less than the others, but the overall pattern was similar between the groups.

Figure 8 shows the difference in transmittance between the right and left corneas of three rabbits (#6, #9, and #11) and the seven rabbits. The solid line represents the rabbits whose corneal thickness differences were less than 7 µm, and the dashed line represents the remaining rabbits. The transmittance difference of the three rabbits was 3%–4% less than the others, but the overall pattern was similar between the groups.

**Figure 9:**
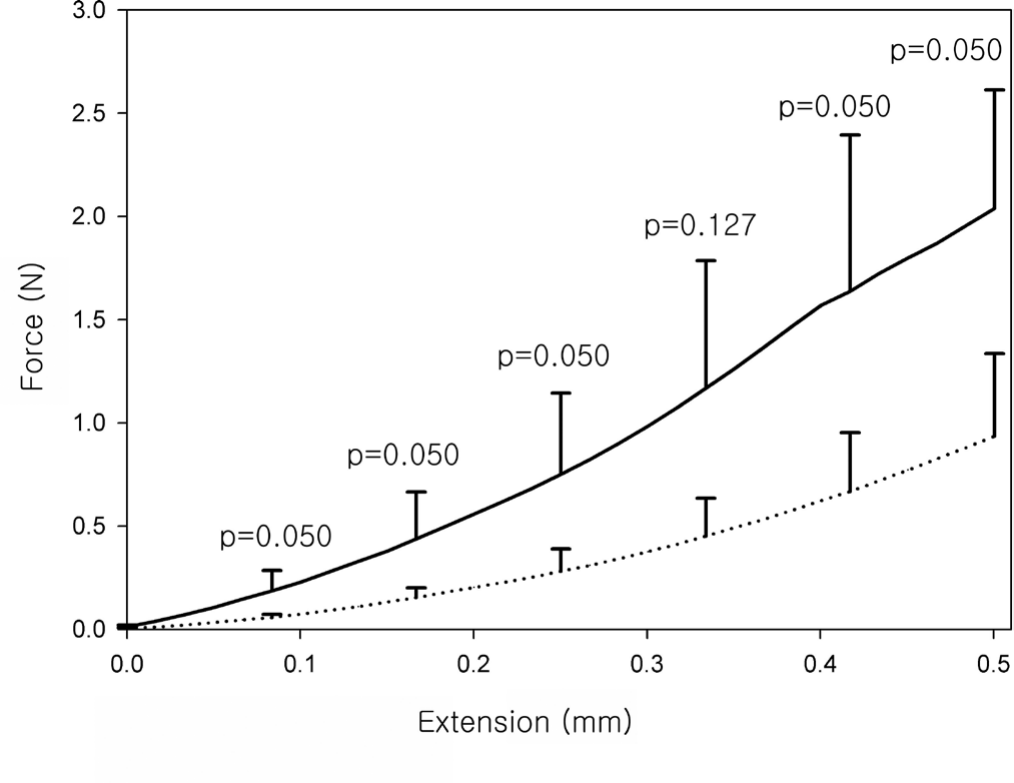
The average force required to extend the cornea over 500 µm in the cross-linking group and the control group. In the control groups (dashed line), extension of the cornea required a gradual and uniform increase in the force. By comparison, greater force was necessary to extend the cross-linked corneas (solid line) over 500 µm. The error bars represent the standard deviation of force between animals in each group.

Figure 9 shows the average force required to extend the cornea over 500 µm in the cross-linking group and the control group. In the control corneas, extending the cornea required a gradual and uniform increase in the force. In comparison, greater force was necessary to extend the cross-linked corneas over 500 µm.

**Figure 10:**
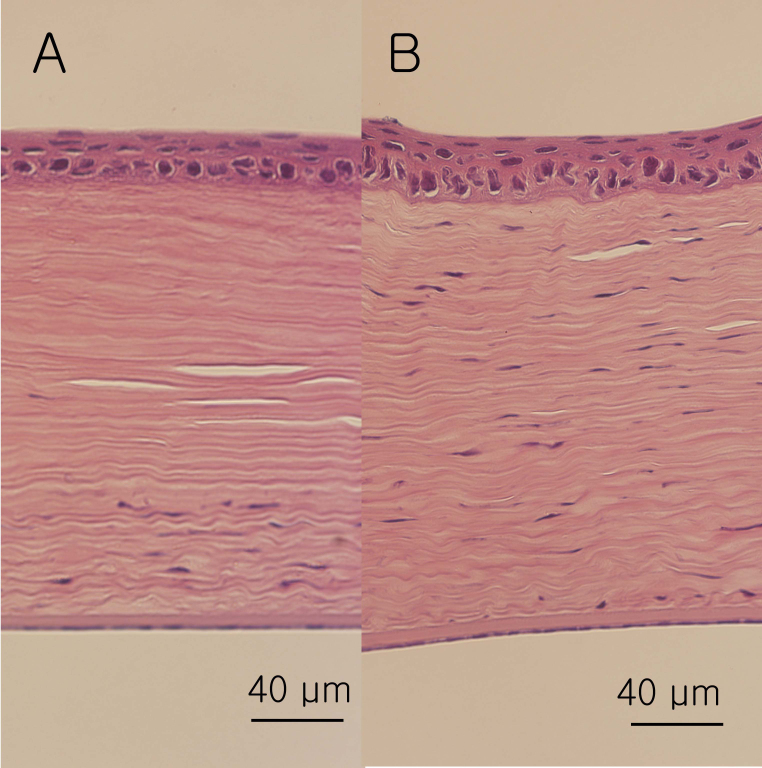
The hematoxylin and eosin–stained images of the cross-linked and control corneas. There were few keratocytes in the anterior stroma of the cross-linked corneas (**A**) compared to the control corneas (**B**). Neither the epithelia nor Descemet’s membrane showed any significant differences (hematoxylin and eosin, 40X).

Figure 10 shows the H&E-stained images of the cross-linked and control corneas. There were few keratocytes in the anterior and middle stroma of the cross-linked corneas compared to the control corneas. The keratocytes in the posterior stroma of the cross-linked corneas were larger than those of the control corneas. We did not find a scar or haze in the stroma of any sections of the cross-linked corneas with high magnification. Neither the epithelium nor Descemet’s membrane showed any significant differences (H&E, 40X).

**Figure 11:**
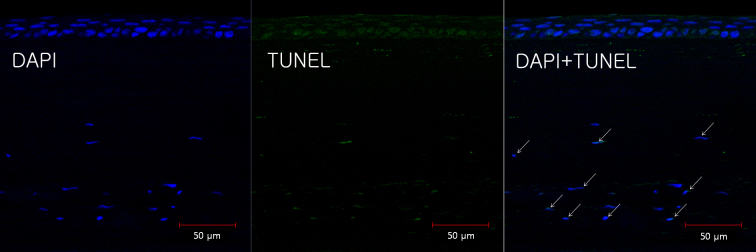
A representative result from terminal deoxynucleotidyl transferase-mediated dUTP nick end labeling staining in the cross-linked corneas. Terminal deoxynucleotidyl transferase-mediated dUTP nick end labeling (TUNEL)-positive apoptotic cells were found at the posterior stroma of the cross-linked corneas.

Figure 11 shows representative results from TUNEL staining in the cross-linked corneas. TUNEL-positive cells were found in the posterior stroma of the cross-linked corneas. There were no TUNEL-positive cells in the control corneas (not shown here).

## Discussion

To our knowledge, this is the first study of the UV-VIS light transmission spectrum of the cornea after cross-linking. In one report, ex vivo cross-linking was performed on enucleated porcine eyes, and the transmission spectrum was measured immediately afterward to evaluate the importance of removing the epithelium in facilitating the entry of riboflavin into the corneal stroma [18]. In another report, regarding the safety of UVA 365 nm itself for the corneal endothelium, the absorption coefficient of UVA 365 nm in postmortem human corneas was measured, after riboflavin was instilled on the corneal surface [19]. However, we performed cross-linking on rabbit corneas in vivo, and after 7 weeks, we measured the UV-VIS light transmission spectrum to determine the effect of cross-linking on the UV-VIS light transmission through the cornea. The experimental protocol was similar to the clinical environment.

We measured the transmission spectrum 7 weeks after cross-linking to avoid the decrease in transmission secondary to cornea edema. In this study, corneal edema was severe on postoperative days 2 and 3. After 1 week, no edema was identifiable with the naked eye. We also confirmed via measuring the central corneal thickness that there was no corneal edema when the transmission was measured. Of course, epithelial healing was complete at the time; thus, the effects of removing the epithelium were avoided. Most of the riboflavin in the corneal stroma would have been absorbed by this time and thus would be unlikely to affect the transmittance spectrum.

In one of the 12 rabbits, corneal neovascularization was found in the cross-linked corneas. In another rabbit, a granular-type central opacity was found. Corneal haze may develop after cross-linking [20,21]. However, to our knowledge there are no reports of neovascularization or granular-type opacities. If these complications develop consistently after cross-linking, the clinical implications will be significant despite the low incidence. We plan to evaluate these complications further.

The central corneal thickness of the cross-linked corneas was smaller than that of the control corneas. This confirmed, indirectly, that cross-linking of the right corneas was successful. In one human study, the corneal thickness decreased until 3 months post-treatment, and then returned to baseline at around 12 months post-treatment [22]. In this study, in seven of ten rabbits, the cross-linked corneal thickness was significantly lower than that of the control corneas. In three rabbits, however, the corneal thickness differences between the cross-linked and control corneas were negligible. We worried that cross-linking might be unsuccessful in these three rabbits. Therefore, we compared the transmission spectra of the seven rabbits in which the cross-linked corneal thickness was significantly lower than that of the controls and the remaining three rabbits. The average transmission difference in the three rabbits was 3%–4% smaller than that of the seven rabbits, but the pattern was similar, which meant cross-linking was successful in these rabbits.

UVA corneal collagen cross-linking leads to an increase in stiffness in porcine corneas and human corneas [23-25]. In this study, the cross-linked corneas were stiffer than the control corneas, suggesting successful cross-linking in the right corneas.

According to the H&E staining, cross-linked corneas compared to the control corneas were different. This is not consistent with a previous report [17]. In one histologic evaluation of rabbit corneas after cross-linking, during the early period after treatment, apoptotic loss of keratocytes in the anterior stroma was evident [17]. Many large spindle-shaped migratory fibroblasts (i.e., activated keratocytes) were observed in the adjacent untreated stroma. However, it resolved completely after 6 weeks. In our research, after 7 weeks, there was still no keratocytes in the anterior stoma. Conversely, in a clinical research study, there was almost complete loss of keratocytes in the anterior stoma in keratoconus patients, 5 to 30 months after treatment [26]. The H&E staining results in our research suggested successful cross-linking.

Riboflavin by itself is not cytotoxic, but as a photosensitizer, it increases the absorption of UVA, which induces the cellular damage [27]. There were some reports about keratocyte apoptosis after cornea cross-linking [28,29]. In a rabbit study, at 24 h, corneas from the UVA-riboflavin cross-linking group had significantly more apoptosis than the UVA alone and riboflavin alone groups [28]. TUNEL-positive cells were observed only occasionally at 4 weeks after surgery in each group. By week 6, the cellular distribution pattern within the stroma was back to normal. However, in the human cornea study, the TUNEL-stained sections of cross-linked corneas showed apoptotic cells in the anterior stroma after 6 months [29]. In this study, we found TUNEL-positive cells in the posterior stroma of the cross-linked corneas after 7 weeks.

We determined the surface irradiance as 3m W/cm^2^ referring to Wollensak’s rabbit experiment [17]. The cytotoxicity level for keratocytes was in the range of 0.49– 0.77 mW/cm^2^ irradiance [30]. However, intensity of ultraviolet decreases rapidly as it passes through the stroma according to Lambert-Beer [30]. Thus, we found most apoptotic cell losses at the anterior and middle stroma unlike the posterior stroma. Ultraviolet light might cause corneal scars, haze, and keratitis at the anterior stroma. However, it is unlikely that ultraviolet light causes inflammation in the eye, especially in the lens, retinal photoreceptor, and retinal pigment epithelium.

In this study, we compared the 300–650 nm transmission spectra of the cross-linked and control corneas. The transmission spectrum pattern of the control corneas was consistent with a previous report [13]. The spectrum of the cross-linked corneas showed patterns similar to those of the control corneas but at a lower overall transmittance. Figure 6 shows the UV-VIS transmittance of the control and cross-linked corneas demonstrating that the spectral transmittance was decreased by cross-linking. In general, although the cross-linked corneas did not show any gross opacity, the transmittance was 10%–20% lower than that of the controls. This may be due to a subtle haze that could not be detected with the naked eye. Corneal haze may develop after cross-linking [20,21].

Two animal studies that performed the alpha smooth muscle actin antibody (α-SMA) stain for myofibroblasts after cross-linking [17,28]. In a rabbit study, there were scattered groups of α-SMA positive myofibrocytes in the corneal stroma adjacent to the treatment area, by weeks 4 and 6 after treatment [17]. In another rabbit study, few SMA-positive cells were detected in the central corneas in the riboflavin-UVA group, at 4 weeks after treatment [28]. In this study, we did not find a scar or haze in the H&E-stained corneas.

However, the difference between the transmission of the control and that of the cross-linked corneas was larger in ultraviolet light than in visible light, and had a maximum at 320 nm (Figure 7). The reason is the maximum reduction in the transmittance of the cross-linked corneas compared with the control corneas occurred at 320 nm. For reference, the wavelength of the light source used in the cross-linking was 365 nm.

What type of collagens protects against UV penetration is important to discover. However, we did not find the information in the current literature. In the human cornea, the basement membrane is composed of collagen type IV collagen. The collagen fibers in Bowman’s layer are primarily collagen types I and III. Collagen in the corneal stroma is mostly type I, with smaller amounts of types III, V, and VI also present. Descemet’s membrane is composed primarily of collagen types IV and VIII [31]. Because the stroma constitutes the largest portion, more than 90%, of the thickness of the cornea, we speculate that type I collagen is the most important for UV penetration. We will investigate what type of collagens protects against UV penetration in the further study.

The difference in the spectral transmittance exhibited a local hump around 425–455 nm and a focal maximum at 350 nm. The former may be due to remnant riboflavin in the stroma. We expected that 7 weeks after the treatment, the riboflavin would be absorbed almost completely. The remnant riboflavin may have caused the local hump in the spectral transmission difference because the maximal absorption is at around 450 nm [18]. However, we could not determine the origin of the focal maximum at 350 nm. There is no literature about the transmittance reduction through the cross-linked cornea at 350 nm. One possibility is changes in keratocytes after UV exposure, including apoptotic cell loss and hyaluronan from repopulating keratocytes [32].

At the beginning of this study, we were concerned about increased ultraviolet transmission after cross-linking. However, the ultraviolet transmission decreased post-procedure, unexpectedly. If the decrease in ultraviolet transmission is larger than that of visible light, as in our data, the procedure may result in protective effects against ultraviolet damage. In this animal experiment, of course, we could not measure visual acuity. Whether a decrease in visible light transmission causes a decrease in corrected visual acuity or it has clinical significance in the human cornea remains to be determined. Our study has the following strengths: First, we measured the transmittance spectra 7 weeks after in vivo cross-linking. Second, we used an epithelium-removed fellow cornea as a control. Third, we confirmed that our cross-linking was successful via pachymeter, tensile force-extension measurement, and histologic evaluation.

In this study, we measured the UV-VIS transmission spectrum of rabbit corneas after cross-linking to determine the effect of UVA cross-linking on the transmission of the UV-VIS. The transmittance of the cross-linked corneas was 10%–20% lower than that of the control corneas. The difference between the transmission of the control and the cross-linked corneas was larger in ultraviolet light than in visible light, and had a maximum at 320 nm. Thus, UV collagen cross-linking showed a protective effect against ultraviolet penetration.

## References

[1] Wollensak G, Spoerl E, Seiler T (2003). Riboflavin/ultraviolet-a-induced collagen crosslinking for the treatment of keratoconus.. Am J Ophthalmol.

[2] Mazzotta C, Balestrazzi A, Traversi C, Baiocchi S, Caporossi T, Tommasi C, Caporossi A (2007). Treatment of progressive keratoconus by riboflavin-UVA-induced cross-linking of corneal collagen: ultrastructural analysis by Heidelberg Retinal Tomograph II in vivo confocal microscopy in humans.. Cornea.

[3] Caporossi A, Baiocchi S, Mazzotta C, Traversi C, Caporossi T (2006). Parasurgical therapy for keratoconus by riboflavin-ultraviolet type A rays induced cross-linking of corneal collagen: preliminary refractive results in an Italian study.. J Cataract Refract Surg.

[4] Wollensak G (2006). Crosslinking treatment of progressive keratoconus: new hope.. Curr Opin Ophthalmol.

[5] Wittig-Silva C, Whiting M, Lamoureux E, Lindsay RG, Sullivan LJ, Snibson GR (2008). A randomized controlled trial of corneal collagen cross-linking in progressive keratoconus: preliminary results.. J Refract Surg.

[6] Raiskup-Wolf F, Hoyer A, Spoerl E, Pillunat LE (2008). Collagen crosslinking with riboflavin and ultraviolet-A light in keratoconus: long-term results.. J Cataract Refract Surg.

[7] Kymionis GD, Diakonis VF, Kalyvianaki M, Portaliou D, Siganos C, Kozobolis VP, Pallikaris AI (2009). One-year follow-up of corneal confocal microscopy after corneal cross-linking in patients with post laser in situ keratosmileusis ectasia and keratoconus.. Am J Ophthalmol.

[8] Caporossi A, Mazzotta C, Baiocchi S, Caporossi T (2010). Long-term results of riboflavin ultraviolet a corneal collagen cross-linking for keratoconus in Italy: the Siena eye cross study.. Am J Ophthalmol.

[9] Wollensak G, Spoerl E, Reber F, Seiler T (2004). Keratocyte cytotoxicity of riboflavin/UVA-treatment in vitro.. Eye (Lond).

[10] Maurice DM (1957). The structure and transparency of the cornea.. J Physiol.

[11] Benedek GB (1971). Theory of transparency of the eye.. Appl Opt.

[12] Farrell RA, McCally RL, Tatham PE (1973). Wave-length dependencies of light scattering in normal and cold swollen rabbit corneas and their structural implications.. J Physiol.

[13] Walsh JE, Bergmanson JP, Koehler LV, Doughty MJ, Fleming DP, Harmey JH (2008). Fibre optic spectrophotometry for the in vitro evaluation of ultraviolet radiation (UVR) spectral transmittance of rabbit corneas.. Physiol Meas.

[14] Freegard TJ (1997). The physical basis of transparency of the normal cornea.. Eye (Lond).

[15] Ghanem RC, Santhiago MR, Berti TB, Thomaz S, Netto MV (2010). Collagen crosslinking with riboflavin and ultraviolet-A in eyes with pseudophakic bullous keratopathy.. J Cataract Refract Surg.

[16] Cejka C, Platenik J, Guryca V, Sirc J, Michalek J, Brunova B, Cejkova J (2007). Light absorption properties of the rabbit cornea repeatedly irradiated with UVB rays.. Photochem Photobiol.

[17] Wollensak G, Iomdina E, Dittert DD, Herbst H (2007). Wound healing in the rabbit cornea after corneal collagen cross-linking with riboflavin and UVA.. Cornea.

[18] Hayes S, O'Brart DP, Lamdin LS, Doutch J, Samaras K, Marshall J, Meek KM (2008). Effect of complete epithelial debridement before riboflavin-ultraviolet-A corneal collagen crosslinking therapy.. J Cataract Refract Surg.

[19] Koppen C, Gobin L, Tassignon MJ (2010). The absorption characteristics of the human cornea in ultraviolet-a crosslinking.. Eye Contact Lens.

[20] Mazzotta C, Balestrazzi A, Baiocchi S, Traversi C, Caporossi A (2007). Stromal haze after combined riboflavin-UVA corneal collagen cross-linking in keratoconus: in vivo confocal microscopic evaluation.. Clin Experiment Ophthalmol.

[21] Raiskup F, Hoyer A, Spoerl E (2009). Permanent corneal haze after riboflavin-UVA-induced cross-linking in keratoconus.. J Refract Surg.

[22] Greenstein SA, Shah VP, Fry KL, Hersh PS (2011). Corneal thickness changes after corneal collagen crosslinking for keratoconus and corneal ectasia: one-year results.. J Cataract Refract Surg.

[23] Wollensak G, Spoerl E, Seiler T (2003). Stress-strain measurements of human and porcine corneas after riboflavin-ultraviolet-A-induced cross-linking.. J Cataract Refract Surg.

[24] Spoerl E, Huhle M, Seiler T (1998). Induction of cross-links in corneal tissue.. Exp Eye Res.

[25] Kohlhaas M, Spoerl E, Schilde T, Unger G, Wittig C, Pillunat LE (2006). Biomechanical evidence of the distribution of cross-links in corneas treated with riboflavin and ultraviolet A light.. J Cataract Refract Surg.

[26] Messmer EM Meyer P Herwig MC Loeffler KU Schirra F Seitz B Thiel M Reinhard T Kampik A Auw-Haedrich C Morphological and Immunohistochemical Changes After Corneal Cross-Linking. Cornea 201332:111-7 22580432 10.1097/ICO.0b013e31824d701b

[27] Cho KS, Lee EH, Choi JS, Joo CK (1999). Reactive oxygen species-induced apoptosis and necrosis in bovine corneal endothelial cells.. Invest Ophthalmol Vis Sci.

[28] Salomão MQ, Chaurasia SS, Sinha-Roy A, Ambrósio R, Esposito A, Sepulveda R, Agrawal V, Wilson SE (2011). Corneal wound healing after ultraviolet-A/riboflavin collagen cross-linking: a rabbit study.. J Refract Surg.

[29] Mencucci R, Marini M, Paladini I, Sarchielli E, Sgambati E, Menchini U, Vannelli GB (2010). Effects of riboflavin/UVA corneal cross-linking on keratocytes and collagen fibres in human cornea.. Clin Experiment Ophthalmol.

[30] Wollensak G, Spoerl E, Wilsch M, Seiler T (2004). Keratocyte apoptosis after corneal collagen cross-linking using riboflavin/UVA treatment.. Cornea.

[31] Nishida T, Saika S. Cornea and Sclera: Anatomy and Physiology. In: Krachmer JH, Mannis MJ, Holland EJ, editors. CORNEA Fundamentals, Diagnosis and Management. Vol 2. China: Elsevier Inc.; 2011.p 10–15.

[32] Podskochy A, Fagerholm P (1998). Cellular response and reactive hyaluronan production in UV-exposed rabbit corneas.. Cornea.

